# TLR2 and endosomal TLR-mediated secretion of IL-10 and immune suppression in response to phagosome-confined *Listeria monocytogenes*

**DOI:** 10.1371/journal.ppat.1008622

**Published:** 2020-07-07

**Authors:** Brittney N. Nguyen, Alfredo Chávez-Arroyo, Mandy I. Cheng, Maria Krasilnikov, Alexander Louie, Daniel A. Portnoy

**Affiliations:** 1 Graduate Group in Microbiology, University of California, Berkeley, Berkeley, California, United States of America; 2 Department of Molecular and Cell Biology, University of California, Berkeley, Berkeley, California, United States of America; 3 Department of Plant and Microbial Biology, University of California, Berkeley, Berkeley, California, United States of America; University of Pennsylvania, UNITED STATES

## Abstract

*Listeria monocytogenes* is a facultative intracellular bacterial pathogen that escapes from phagosomes and induces a robust adaptive immune response in mice, while mutants unable to escape phagosomes fail to induce a robust adaptive immune response and suppress the immunity to wildtype bacteria when co-administered. The capacity to suppress immunity can be reversed by blocking IL-10. In this study, we sought to understand the host receptors that lead to secretion of IL-10 in response to phagosome-confined *L*. *monocytogenes* (Δ*hly*), with the ultimate goal of generating strains that fail to induce IL-10. We conducted a transposon screen to identify Δ*hly L*. *monocytogenes* mutants that induced significantly more or less IL-10 secretion in bone marrow-derived macrophages (BMMs). A transposon insertion in *lgt*, which encodes phosphatidylglycerol-prolipoprotein diacylglyceryl transferase and is essential for the formation of lipoproteins, induced significantly reduced IL-10 secretion. Mutants with transposon insertions in *pgdA* and *oatA*, which encode peptidoglycan N-acetylglucosamine deacetylase and O-acetyltransferase, are sensitive to lysozyme and induced enhanced IL-10 secretion. A Δ*hly*Δ*pgdA*Δ*oatA* strain was killed in BMMs and induced enhanced IL-10 secretion that was dependent on Unc93b1, a trafficking molecule required for signaling of nucleic acid-sensing TLRs. These data revealed that nucleic acids released by bacteriolysis triggered endosomal TLR-mediated IL-10 secretion. Secretion of IL-10 in response to infection with the parental strain was mostly TLR2-dependent, while IL-10 secretion in response to lysozyme-sensitive strains was dependent on TLR2 and Unc93b1. In mice, the IL-10 response to vacuole-confined *L*. *monocytogenes* was also dependent on TLR2 and Unc93b1. Co-administration of Δ*hly* and Δ*actA* resulted in suppressed immunity in WT mice, but not in mice with mutations in Unc93b1. These data revealed that secretion of IL-10 in response to *L*. *monocytogenes* infection *in vitro* is mostly TLR2-dependent and immune suppression by phagosome-confined bacteria *in vivo* is mostly dependent on endosomal TLRs.

## Author summary

*Listeria monocytogenes* is a Gram-positive bacterial pathogen that has shown promise as a vaccine-delivery vector because of its ability to stimulate a robust T-cell response. The efficacy of a vaccine is in part tied to how well it avoids inducing the immunosuppressive cytokine IL-10. In this work, we investigated the bacterial and host factors that contribute to secretion of IL-10 and immunosuppression following infection with a strain of *L*. *monocytogenes* that cannot escape from host cell vacuoles. We identified TLR2 and Unc93b1-dependent nucleic-acid-sensing Toll-like receptors as the primary host mediators of IL-10 secretion and immunosuppression. Unc93b1-dependent TLRs likely recognize nucleic acids released upon bacterial lysis in phagosomes. Strategies that reduce lytic death of bacteria and subsequent recognition of nucleic acids by endosomal TLRs could be used to improve bacteria-based vaccines.

## Introduction

*Listeria monocytogenes* is a Gram-positive facultative intracellular pathogen that has been widely used as a model to study host immune responses. Infection of mice with *L*. *monocytogenes* induces the generation of adaptive immune responses that are protective against subsequent infection and are largely mediated by CD8^+^ T cells [1,2]. Many innate immune factors contribute to control of primary *L*. *monocytogenes* infection. The factors that contribute to development of functional CD8^+^ effector and memory T cells are less clear [3–6]. It has been posited that intracytosolic growth of *L*. *monocytogenes* is a prerequisite for the induction of T cell-mediated immunity because Δ*hly L*. *monocytogenes*, which does not produce the virulence factor listeriolysin O and cannot escape phagocytic vacuoles, fails to induce robust protective immunity [7,8]. However, in 2009 Bahjat *et al*. provided evidence that Δ*hly L*. *monocytogenes* fails to induce robust protective immunity because it induces secretion of IL-10 early during infection. When IL-10 signaling was inhibited by administration of anti-IL-10 receptor blocking antibody, the protective capacity of Δ*hly L*. *monocytogenes* was enhanced [9]. Thus, strains that escape phagocytic vacuoles and grow in the cytosol induce generation of protective immunity in part because they avoid inducing IL-10-mediated suppression.

IL-10 is an anti-inflammatory cytokine that acts on many cell types to downregulate inflammation and recruitment of immune cells, thereby limiting immunopathology during the resolution of an immune response [10]. Infection of wildtype mice with *L*. *monocytogenes* results in high levels of serum IL-10 3–4 days post infection [11,12]. Mice lacking IL-10 clear *L*. *monocytogenes* faster than wildtype mice, indicating that IL-10 partially suppresses the primary innate immune response to *L*. *monocytogenes* [13,14]. Importantly, though IL-10 is expressed following primary WT *L*. *monocytogenes* infection, WT infection still results in the generation of a robust protective immune response.

IL-10 is secreted by many cell types, including macrophages, dendritic cells (DCs), neutrophils, NK cells, T-regulatory cells and B cells [10]. The IL-10 present in serum 3 to 4 days after WT *L*. *monocytogenes* infection of mice is mostly derived from NK cells [11]. However, Δ*hly L*. *monocytogenes* induces IL-10 secretion that is detectable four hours after infection, and likely comes from macrophages or dendritic cells, which are the first cells infected by *L*. *monocytogenes* in the spleen [9,15,16]. In macrophages, IL-10 expression can be triggered by activation of the pattern recognition receptors TLR2, TLR3, TLR4, and TLR9 [10,17]. Macrophages secrete high levels of IL-10 in response to the TLR9 agonist CpG, while myeloid DCs secrete much less [17]. Myeloid DCs can secrete IL-10 upon activation of TLR2, TLR4, TLR9, and also the C-type lectins DC-SIGN and Dectin-1 [10,18–20]. Thus, there are diverse pathways leading to IL-10 secretion. The timing of IL-10 secretion and cell types that respond impacts the generation of adaptive immune responses [21].

The bacterial components and signaling pathways that lead to induction of IL-10 following infection with Δ*hly L*. *monocytogenes* have not been experimentally addressed. In this study, we conducted a genetic screen to identify the components of vacuole-confined Δ*hly L*. *monocytogenes* that induce IL-10 secretion from murine bone marrow-derived macrophages. We investigated the host signaling pathways that lead to recognition of Δ*hly L*. *monocytogenes* and secretion of IL-10 in macrophages and mice, and how these host signaling pathways affect vaccination and immune suppression.

## Results

### Genetic screen to identify *L*. *monocytogenes* mutants that induce enhanced or diminished levels of IL-10

**Fig 1:**
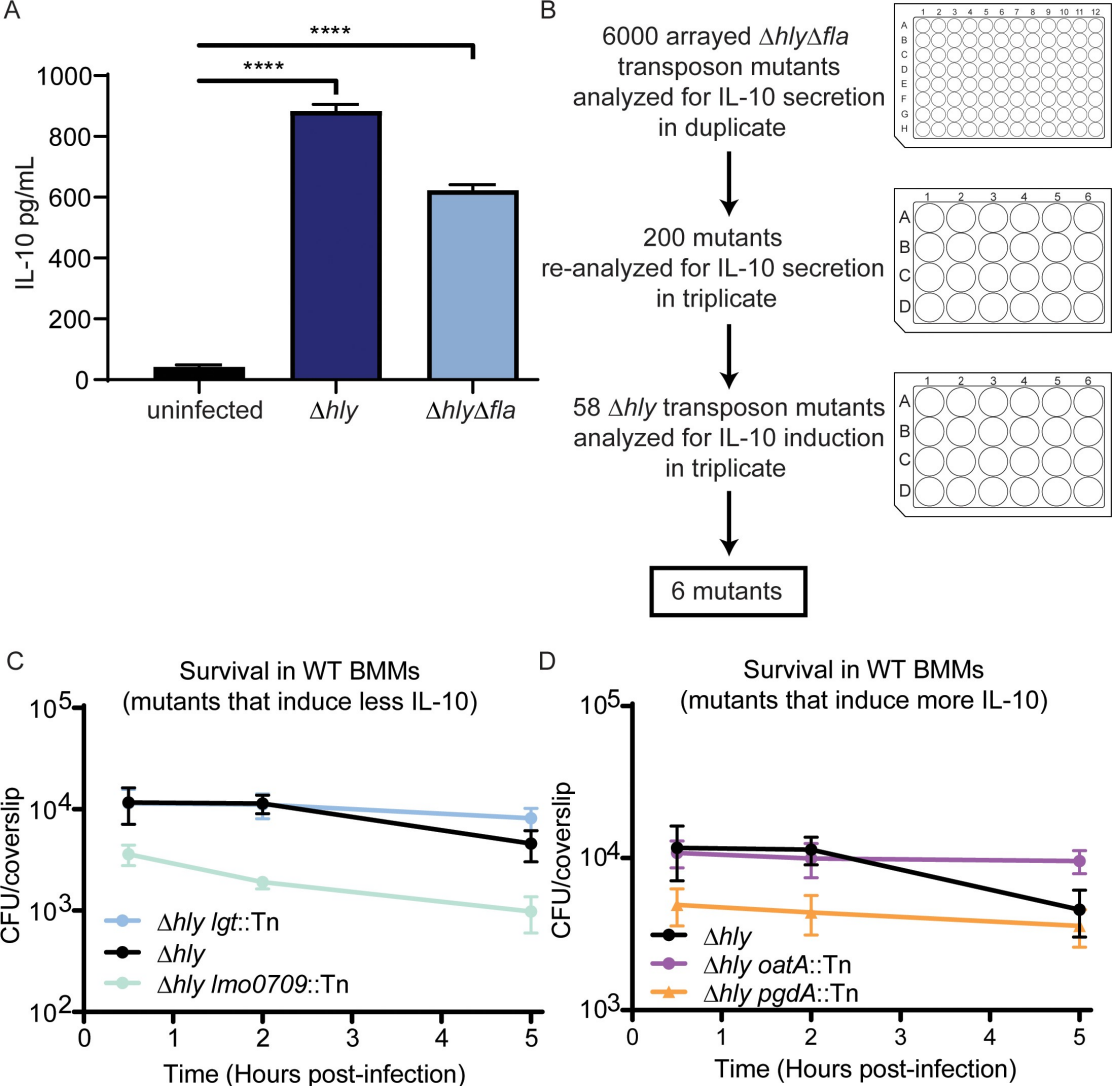
Vacuole-confined *L*. *monocytogenes* induce IL-10 secretion from BMMs. (A) BMMs were infected with *L*. *monocytogenes* lacking LLO (Δ*hly*) or lacking LLO and flagellin (Δ*hly*Δ*fla*) for four hours. Cell supernatant was collected and IL-10 was measured by ELISA. Data shown are representative of two independent experiments. Mean and SD are shown. n = 3. Data analyzed using Holm-Sidak’s multiple comparisons test. (B) Schematic of screen. 6000 Δ*hly*Δ*fla* transposon mutants were analyzed for enhanced or diminished IL-10 expression in duplicate in a 96-well plate format. 200 mutants were selected for secondary screening, and were analyzed for IL-10 in triplicate in a 24-well plate format. The mutations from 58 mutants that induced significantly enhanced or diminished IL-10 were transduced into a Δ*hly* background and analyzed for IL-10 secretion. Six mutations that resulted in enhanced or diminished IL-10 secretion were identified. (C and D) Survival of transposon mutants was quantified in BMMs. Mean and SEM are shown. Data are pooled from three coverslips per experiment for three independent experiments.

The goal of this study was to identify *L*. *monocytogenes* determinants that contribute to induction of IL-10 secretion from BMMs. We screened a library of Δ*hly* transposon mutants for their ability to induce IL-10 secretion from BMMs. The transposon library was generated in a flagellin-negative Δ*hly* background (Δ*hly*Δ*fla*) to eliminate the identification of false low-IL-10 mutants resulting from mutations in flagellar components that reduce infection efficiency. Previous work demonstrated that infection of mice with Δ*hly* is immunosuppressive and IL-10 levels are increased in the serum four hours post-infection [9], while infection with WT leads to IL-10 that peaks 3–4 days post-infection [12,13]. To determine whether significant IL-10 is secreted by *L*. *monocytogenes*-infected BMMs four hours post-infection, BMMs were infected with Δ*hly* and Δ*hly*Δ*fla L*. *monocytogenes*, and IL-10 was measured from cell supernatants by ELISA. BMMs infected with both Δ*hly* and Δ*hly*Δ*fla L*. *monocytogenes* secreted significantly more IL-10 than uninfected BMMs four hours post-infection (Fig 1A). As *L*. *monocytogenes* infection leads to significant expression of IL-10 in both BMMs and mice four hours post-infection, we analyzed cell supernatants for IL-10 levels four hours post-infection in our screen.

BMMs were infected in duplicate with 6000 gridded Δ*hly*Δ*fla* transposon mutants in 96-well plates (Fig 1B). 200 mutants that induced enhanced or diminished IL-10 levels compared to Δ*hly*Δ*fla* were selected for secondary screening. For secondary screening, optical density of bacterial cultures was adjusted to eliminate differences in IL-10 secretion due to growth differences in broth. 59 of the 200 mutants induced significantly enhanced or diminished IL-10 levels compared to Δ*hly*Δ*fla* in a 24-well format (S1 Table). The transposons in 58 mutants were phage-transduced from the Δ*hly*Δ*fla* background into a Δ*hly* background. In the Δ*hly* background, only two mutants induced less IL-10 secretion and four mutants induced increased IL-10 secretion (Table 1).

**Table 1:** IL-10 secretion four hours post-infection of BMMs with deletion mutants and transposon mutants identified in genetic screen.

**Diminished IL-10**	**Gene Annotation**	**IL-10 (% Δ*hly*)**	**Strain Number**
Δ*lgt*	*lgt*	30	DP-L7003
*Lmo2482*::Tn	*lgt*	27	DP-L7111
*Lmo0709*::Tn	hypothetical protein	41	DP-L7080
**Enhanced IL-10**	**Gene Annotation**	**IL-10 (% Δ*hly*)**	**Strain Number**
Δ*pgdA*Δ*oatA*	*pgdA/oatA*	263	DP-L7004
*Lmo0415*::Tn	*pgdA*	177	DP-L7074
*Lmo2529*::Tn	ATP synthase F0F1 subunit beta	158	DP-L7112
*Lmo2634*::Tn	*ecfT*	140	DP-L7116
*Lmo1291*::Tn	*oatA*	139	DP-L7089

Notably, a mutant with a transposon insertion in phosphatidylglycerol-prolipoprotein diacylglyceryl transferase (*lgt*), induced nearly no IL-10 secretion, though it entered cells and survived in cells similar to Δ*hly* (Fig 1C). Lgt catalyzes the transfer of a lipid moiety from phosphatidylglycerol onto a cysteine residue of prolipoproteins [22–24]. The resulting lipoproteins are well-known TLR2 agonists [25]. Of the two mutants that induced reduced IL-10 secretion, we focused on understanding the contribution of *lgt* to the induction of IL-10 secretion from BMMs because the other mutant, which had a transposon insertion in lmo0709, had reduced infection capability (Fig 1C).

**Fig 2:**
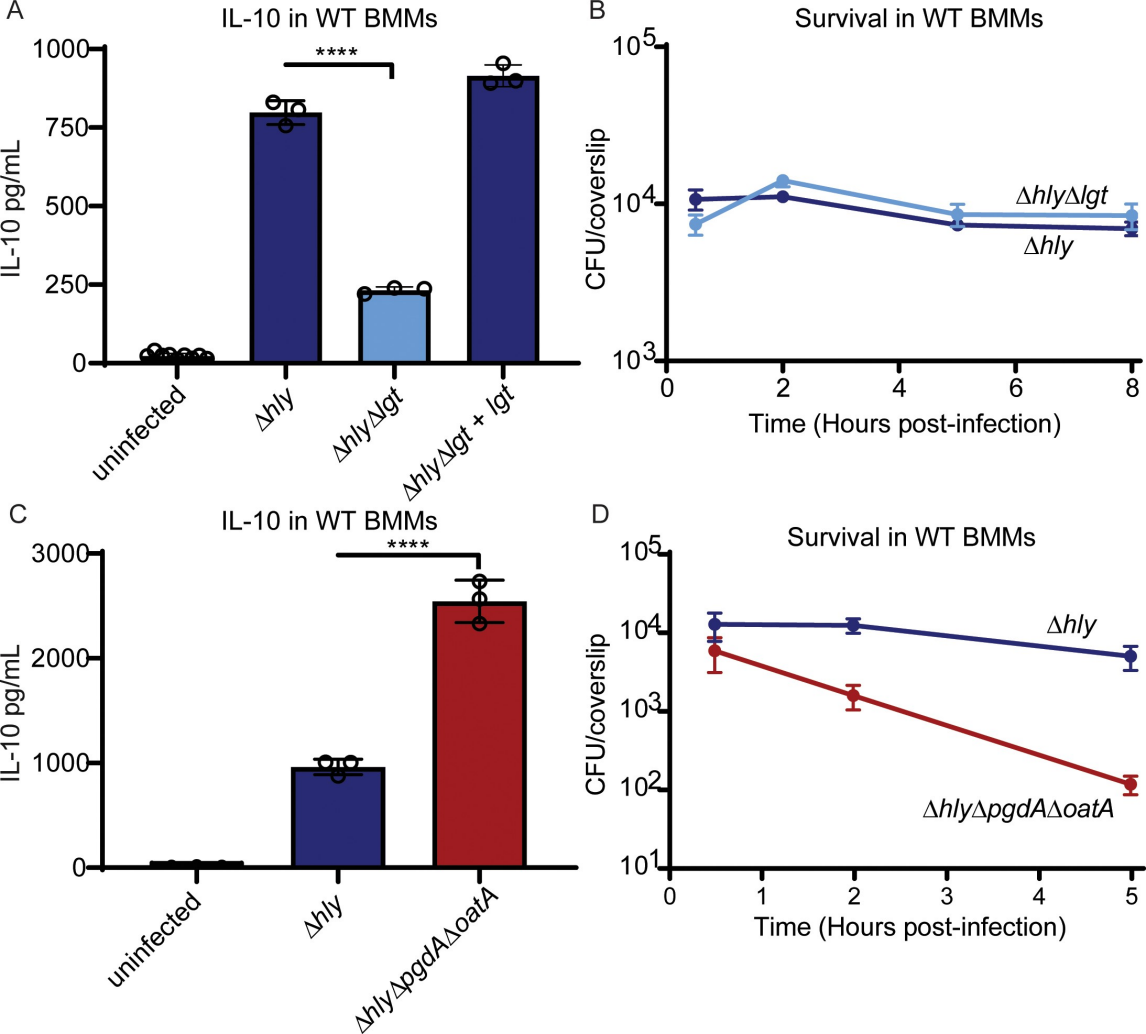
Induction of IL-10 is mediated by lipoproteins and bacteriolysis. (A) IL-10 secretion from BMMs four hours-post infection. Mean and SD are shown. Data are representative of two independent experiments. Data analyzed using Holm-Sidak’s multiple comparison’s test. (B) Survival of *lgt* deletion mutant in BMMs. Data is representative of two independent experiments. Mean and SEM are shown. (C) IL-10 secretion from BMMs four hours-post infection. Mean and SD are shown. Data are representative of three independent experiments. Data analyzed using Holm-Sidak’s multiple comparison’s test. (D) Survival of Δ*hlyΔpgdAΔoatA* mutant in BMMs. Data are pooled from three independent experiments. Mean and SEM are shown.

To confirm the role of *lgt* in the reduced IL-10 phenotype, an in-frame deletion of *lgt* was generated in a Δ*hly* background. Deletion of *lgt* did not affect infection efficiency or survival in BMMs (Fig 2B). An l*gt*-deletion mutant induced significantly reduced IL-10 secretion from WT BMMs compared to Δ*hly*. IL-10 levels were restored when *lgt* was complemented in Δ*hly L*. *monocytogenes* under control of the constitutively active pHyper promoter (Fig 2A). These data indicated that Δ*hly L*. *monocytogenes* primarily induces IL-10 secretion in a lipoprotein- and TLR2-dependent manner.

Of the mutants that induced increased IL-10 secretion, mutations in two of the genes, *pgdA* and *oatA*, have previously been shown to render bacteria more susceptible to lysozyme-mediated killing [26]. We hypothesized that these mutants had increased bacterial lysis within host phagosomes, leading to greater activation of endosomal nucleic acid-sensing TLRs. Though the mutations did not contribute to a noticeable survival defect individually (Fig 1D), a strain lacking both *pgdA* and *oatA* (Δ*hly*Δ*pgdA*Δ*oatA*) induced significantly more IL-10 than Δ*hly* and had a significant survival defect in BMMs (Fig 2C and 2D), indicative of bacterial death in vacuoles. We hypothesized that lysis of bacteria in the phagocytic vacuole could result in release of bacterial nucleic acids and activation of nucleic acid-sensing TLRs.

### IL-10 secretion in BMMs

**Fig 3:**
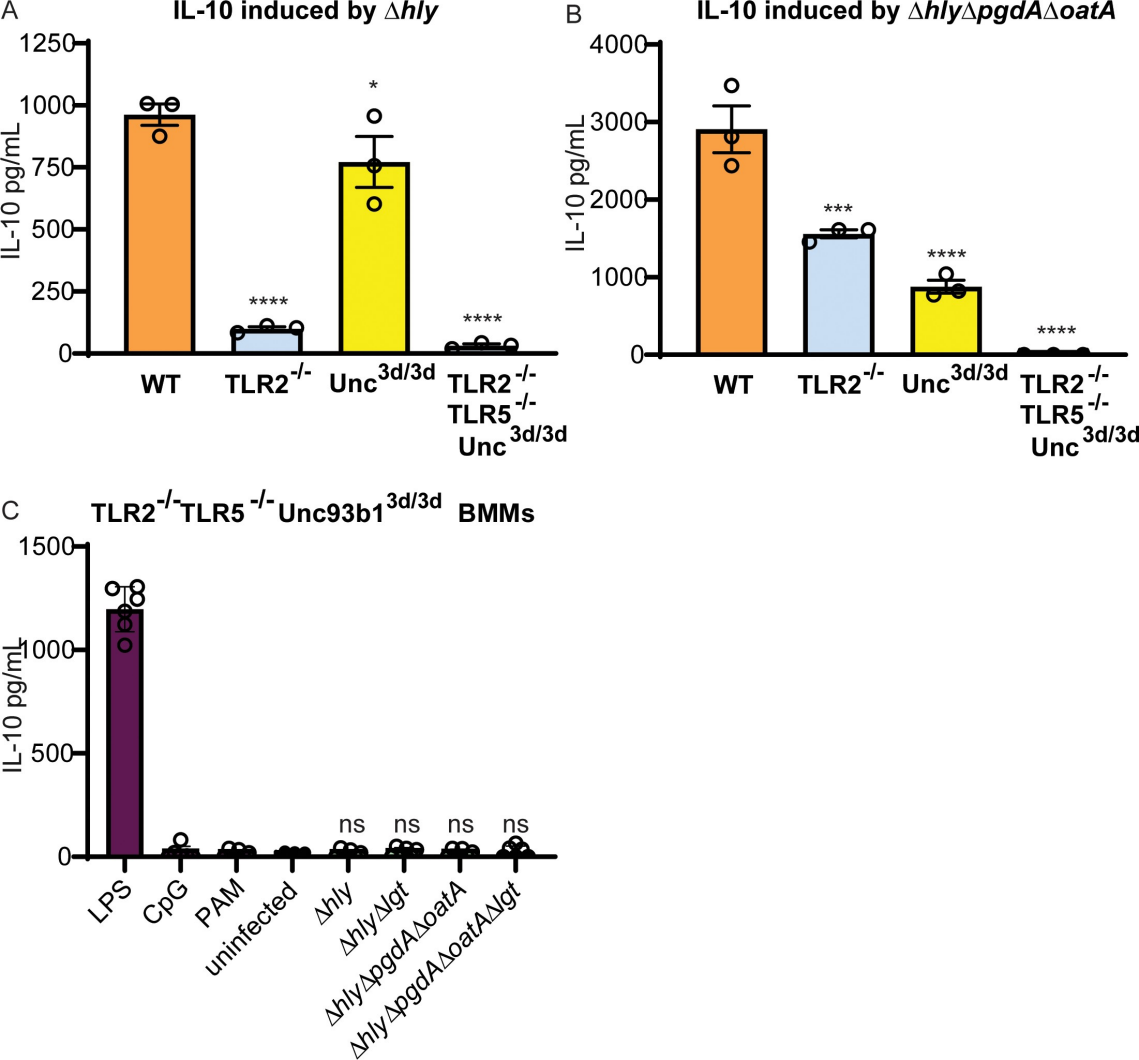
IL-10 secretion from BMMs in response to *L*. *monocytogenes* infection requires TLR2 and endosomal TLR signaling. (A) WT, TLR2^-/-^, Unc93b1^3d/3d^, and TLR2^-/-^TLR5^-/-^Unc93b1^3d/3d^ BMMs were infected with Δ*hly L*. *monocytogenes*. Cell supernatant was harvested four hours post infection and IL-10 was measured by ELISA. Data shown are representative of two independent experiments. Means were compared to WT using Holm-Sidak’s Multiple Comparisons test. (B) WT, TLR2^-/-^, Unc93b1^3d/3d^, and TLR2^-/-^TLR5^-/-^Unc93b1^3d/3d^ BMMs were infected with Δ*hly*Δ*pgdA*Δ*oatA L*. *monocytogenes*. Cell supernatant was harvested four hours post infection and IL-10 was measured by ELISA. Data shown are representative of two independent experiments. Means were compared to WT using Holm-Sidak’s Multiple Comparisons test. (C) TLR2^-/-^TLR5^-/-^Unc93b1^3d/3d^ BMMs were infected with the indicated strains of *L*. *monocytogenes*, or treated with lipopolysaccharide (LPS), a TLR4 agonist; CpG ODN 1668 (CpG), a TLR9 agonist; or Pam2CSK4 (PAM), a TLR2 agonist. Data shown are pooled from two independent experiments. Data analyzed using Holm-Sidak’s Multiple Comparisons test.

While infection with Δ*hly* induced significant IL-10 secretion from WT BMMs, Δ*hly* induced almost no detectable IL-10 secretion in TLR2^-/-^ BMMs (Fig 3A), suggesting that bacterial lipoproteins are the major activators of IL-10 secretion in response to infection with Δ*hly*. In contrast, Δ*hly*Δ*pgdA*Δ*oatA* induced significant levels of IL-10 secretion in TLR2^-/-^ BMMs (Fig 3B), suggesting that bacteriolysis can stimulate a second pathway of IL-10 induction. To determine whether this second pathway was dependent on endosomal nucleic-acid sensing TLRs, we infected BMMs with a mutation in the endosomal TLR trafficking protein Unc93b1, which is essential for endosomal TLR signaling [27]. In cells from mice that have a mutation in Unc93b1, nucleic acid-sensing TLRs do not traffic properly from the endoplasmic reticulum to endolysosomes. As a result, signaling through nucleic acid-sensing TLRs is abrogated, but other TLRs retain normal signaling [28,29]. Δ*hly* induced similar amounts of IL-10 in WT and Unc93b1^3d/3d^ BMMs (Fig 3A), whereas Δ*hly*Δ*pgdA*Δ*oatA* induced significantly less IL-10 secretion in Unc93b1^3d/3d^ BMMs (Fig 3B).

To confirm that IL-10 secretion in response to vacuole-confined *L*. *monocytogenes* resulted from signaling through TLR2 and endosomal TLRs, we infected BMMs with mutations in both pathways (TLR2^-/-^TLR5^-/-^Unc93b1^3d/3d^). These macrophages secreted IL-10 in response to LPS, which is a TLR4 ligand, but not in response to the *L*. *monocytogenes* mutants that were tested (Fig 3C). These results confirmed that IL-10 secretion from BMMs results from signaling through TLR2 and endosomal TLRs.

### Cytokine secretion in mice

**Fig 4:**
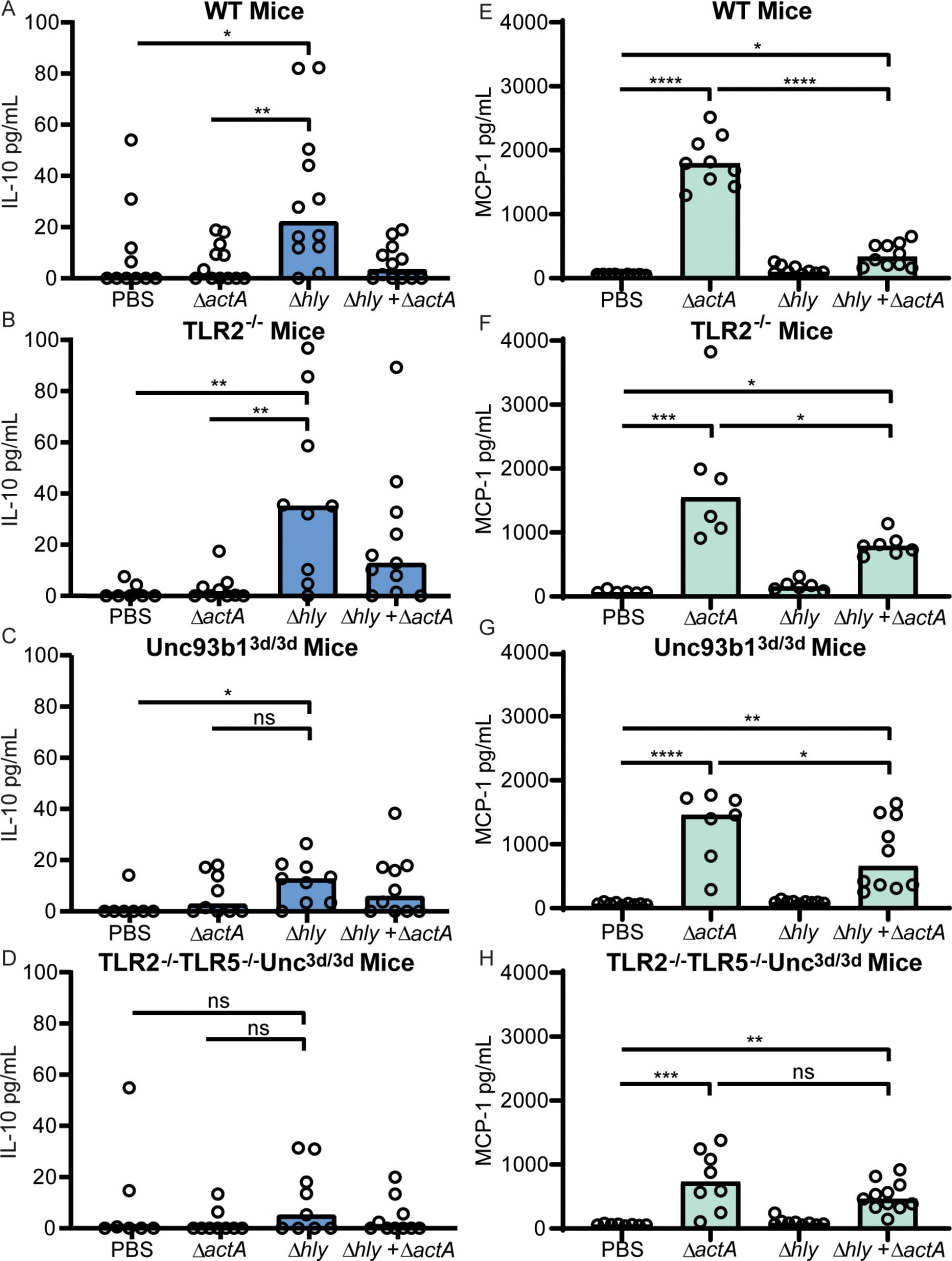
*L*. *monocytogenes* induces IL-10 and MCP-1 secretion in mice. Mice were infected with 10^8^ CFU of Δ*hly*, 10^5^ CFU of Δ*actA*, or a combination of 10^8^ CFU of Δ*hly* and 10^5^ CFU of Δ*actA*. WT C57BL/6J mice (A and E), TLR2^-/-^ (B and F), Unc93b1^3d/3d^ (C and G), and TLR2^-/-^TLR5^-/-^Unc93b1^3d/3d^ (D and H) were infected. Serum measurements of IL-10 four hours post-infection (A-D) and MCP-1 twenty-four hours post-infection (E-H). Data are pooled from two to four independent experiments. Bar represents the median. Data analyzed using Holm-Sidak’s Multiple Comparisons test.

To determine whether secretion of IL-10 in mice was dependent on TLR2 and/or endosomal TLRs, mice were infected intravenously and serum IL-10 was quantified four hours post-infection (Fig 4A–4D). Infection of WT mice with Δ*hly* resulted in significant levels of IL-10, but unlike in BMMs Δ*hly* also induced significant amounts of IL-10 in TLR2^-/-^ mice (Fig 4B), suggesting that bacterial lipoproteins were not the dominant IL-10-inducing molecules in mice. In contrast, IL-10 secretion following Δ*hly L*. *monocytogenes* infection was reduced in Unc93b1^3d/3d^ mice and almost undetectable in TLR2^-/-^TLR5^-/-^Unc93b1^3d/3d^ mice (Fig 4C and 4D). Together, these results indicate that endosomal TLRs were the primary mediators of IL-10 secretion in response to Δ*hly* infection in mice, but that TLR2 may also contribute to induction of IL-10.

One function of IL-10 signaling is to limit proinflammatory cytokine secretion. To investigate the relationship between TLRs and proinflammatory cytokine signaling, serum levels of MCP-1 were quantified 24 hours post-infection (Fig 4E–4H). In WT mice, Δ*hly* did not induce secretion of MCP-1, while Δ*actA*, a strain of *L*. *monocytogenes* that escapes the phagocytic vacuole and replicates in the host cell cytosol, induced significant amounts of MCP-1 (Fig 4E). Likewise, Δ*actA*, but not Δ*hly*, induced significant MCP-1 secretion in TLR2^-/-^ mice (Fig 4F). Because early IL-10 expression is thought to limit later expression of proinflammatory cytokines, we hypothesized that Δ*hly* would induce proinflammatory cytokines in Unc93b1^3d/3d^ and TLR2^-/-^TLR5^-/-^Unc93b1^3d/3d^ mice. However, MCP-1 was not induced in response to Δ*hly* background strains in Unc93b1^3d/3d^ and TLR2^-/-^TLR5^-/-^Unc93b1^3d/3d^ mice (Fig 4G and 4H). These results indicated that endosomal TLR signaling may be required for Δ*hly* to induce a proinflammatory cytokine response.

Vaccination of mice with Δ*actA* has previously been shown to result in high levels of proinflammatory cytokines, whereas vaccination of mice with Δ*hly* induced high levels of IL-10 and low levels of proinflammatory cytokines in WT mice. Strikingly, co-administration of Δ*actA* and Δ*hly* resulted in high levels of IL-10 and low levels of proinflammatory cytokines in WT mice [9], suggesting that Δ*hly* suppresses the proinflammatory responses normally induced by Δ*actA*. We analyzed the levels of MCP-1 in serum as a measure of the proinflammatory cytokine response. It was previously reported that Δ*actA* induced significant amounts of MCP-1 in WT mice, but that co-administration of Δ*actA* with 1000-fold more Δ*hly* CFU resulted in reduced expression of MCP-1 [9]. We observed that Δ*actA* induced significant amounts of MCP-1 in WT mice, and that co-administration of Δ*actA* and Δ*hly* resulted in low levels of MCP-1 secretion, consistent with previous findings (Fig 4E). MCP-1 levels following co-administration of Δ*actA* and Δ*hly* were partially restored to Δ*actA* alone levels in TLR2^-/-^ and Unc93b1^3d/3d^ mice, suggesting that the TLR2 and endosomal TLR signaling pathways contribute to suppression of proinflammatory cytokines (Fig 4F and 4G). In TLR2^-/-^TLR5^-/-^Unc93b1^3d/3d^ mice (Fig 4H), co-administration of Δ*actA* and Δ*hly* resulted in levels of MCP-1 secretion similar to those induced by Δ*actA* alone. These results indicate that suppression of proinflammatory cytokines by Δ*hly* is mediated by TLR2 and Unc93b1-dependent TLRs.

### Suppression of adaptive immunity

**Fig 5:**
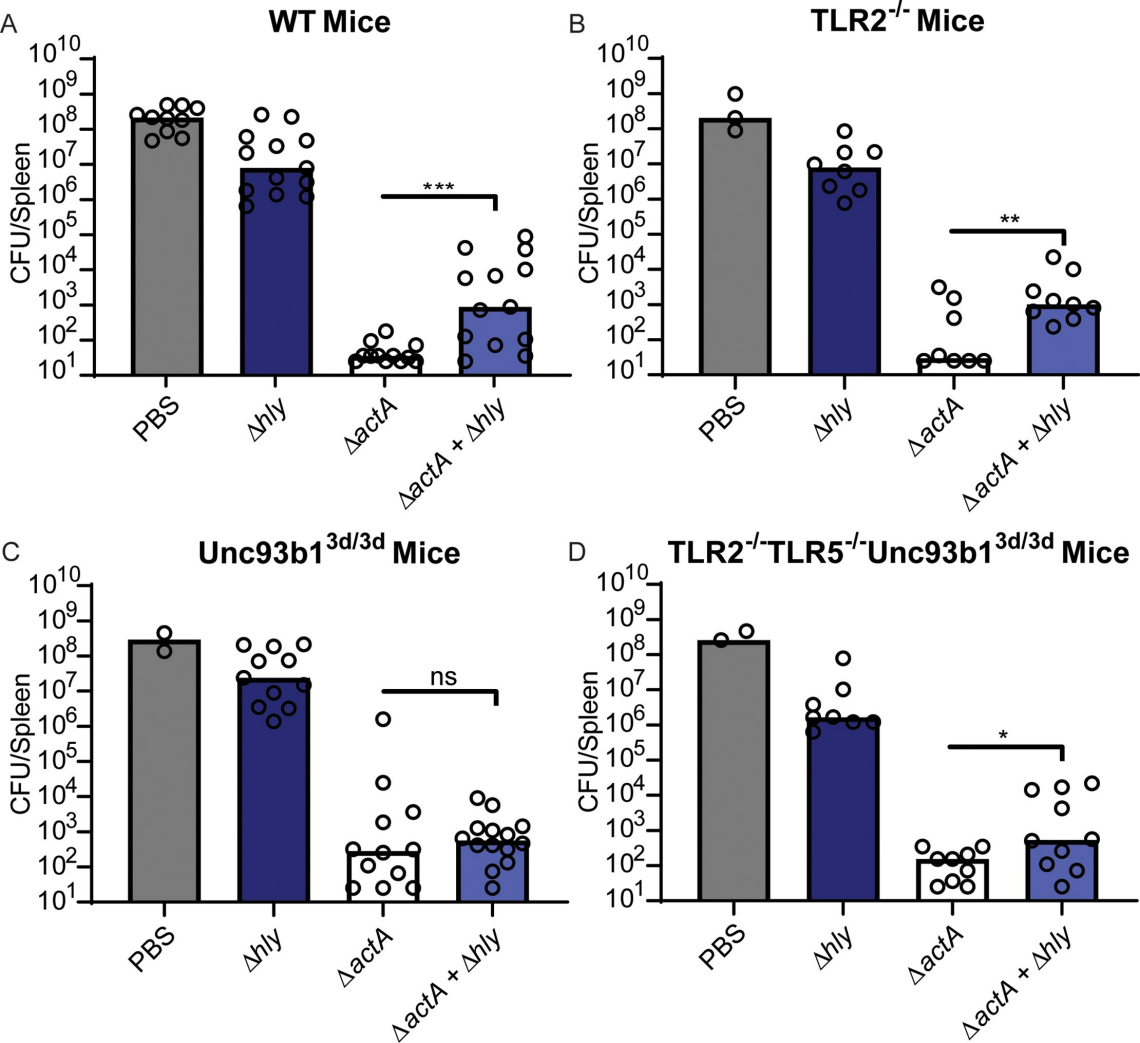
Immune suppression is primarily mediated by endosomal TLR signaling. Mice were infected with 10^8^ CFU of Δ*hly* background strains or 10^5^ CFU of Δ*actA*. WT C57BL/6J mice (A), TLR2^-/-^ (B), Unc93b1^3d/3d^ (C), and TLR2^-/-^TLR5^-/-^Unc93b1^3d/3d^ (D) were infected. Eight weeks post-vaccination, mice were challenged with 5x10^5^ WT *L*. *monocytogenes*. CFU from the spleen were enumerated three days post-challenge. Data are pooled from two to four independent experiments. Bar represents the median. Data analyzed using an unpaired t test.

To investigate the relationship between IL-10 signaling pathways and suppression of adaptive immunity, co-vaccination experiments were performed in which Δ*actA* and 1000-fold more Δ*hly* CFU were injected simultaneously as described [9] (Fig 5). In WT mice, vaccination with Δ*actA* led to the induction of robust adaptive immunity that nearly cleared a subsequent lethal dose of WT *L*. *monocytogenes*. However, the immunity induced by Δ*actA* was suppressed two-logs by co-administration of Δ*hly* (Fig 5A). This suppression was previously shown to depend on IL-10 [8]. We hypothesized that Δ*hly* would suppress immunity in WT and TLR2^-/-^ mice, in which we observed significant secretion of IL-10 and low levels of MCP-1, but not in Unc93b1^3d/3d^ or TLR2^-/-^TLR5^-/-^Unc93b1^3d/3d^ mice, in which we observed no IL-10 and high levels of MCP-1. That was indeed the case, as we observed 2-logs of immune suppression in WT and TLR2^-/-^ mice (Fig 5A and 5B). However, in Unc93b1^3d/3d^ mice co-administration of Δ*actA* with Δ*hly* did not significantly reduce protective immunity (Fig 5C). In TLR2^-/-^TLR5^-/-^Unc93b1^3d/3d^ mice, we observed a 4-fold reduction in protective immunity (Fig 5D).

## Discussion

The results of this study demonstrate that the secretion of IL-10 by BMMs is largely mediated by TLR2-detection of bacterial lipoproteins. However, analysis of mutants that induced enhanced IL-10 revealed a second pathway that was dependent on triggering of Unc93b1-dependent nucleic-acid sensing TLRs in phagosomes by bacteriolysis. In mice, TLR2 and nucleic acid-sensing TLRs contributed to IL-10 secretion.

The results of our analysis of BMMs indicates that TLR2 and Unc93b1-dependent TLRs represent two independent pathways that lead to IL-10 expression. However, our data in mice indicates that TLR2-signaling does not contribute to immune suppression to the same extent as endosomal TLRs. TLR2-deficient mice expressed significant IL-10 following infection with Δ*hly* (Fig 4B). In contrast, infection of Unc93b1^3d/3d^ mice resulted in significant but reduced IL-10 levels compared to WT mice (Fig 4C). Only TLR2^-/-^TLR5^-/-^Unc93b1^3d/3d^ mice failed to express significant IL-10 after Δ*hly* infection (Fig 4D). The reduction in IL-10 correlated with a reduction in immunosuppression, indicating that although TLR2 and endosomal TLRs mediate IL-10 induction, endosomal TLRs may be the major mediators of immune suppression.

The differences in TLR contributions to IL-10 expression in BMMs compared to mice likely reflects differences in their capacity to kill and degrade *L*. *monocytogenes*, but may also reflect differences in TLR expression levels. It is possible that *L*. *monocytogenes-*infected cells in mice express less TLR2 than BMMs. For example, mouse peritoneal macrophages express very low levels of surface TLR2 compared to BMMs [30]. Reduced TLR2 expression on *L*. *monocytogenes-*infected cells in mice would explain why we observed a smaller contribution of TLR2 to IL-10 expression in mice. Additionally, increased bacterial killing in mice likely explains the greater contribution of endosomal TLRs to IL-10 expression in mice compared to BMMs. In mice, CD169+ macrophages, are thought to be the first cells to capture *L*. *monocytogenes* in the spleen and restrict their multiplication [15,31]. In BMMs, which are differentiated and cultured *ex vivo*, Δ*hly L*. *monocytogenes* cannot grow but are not efficiently killed (Fig 2B). In BMMs, in the absence of bacterial killing bacterial lipoproteins are the most abundant TLR stimulus. However, bacterial mutants that had increased lysis in BMMs induced Unc93b1-dependent IL-10 secretion. In contrast to BMMs, peritoneal macrophages taken directly from mice kill vacuole-confined bacteria, suggesting that *L*. *monocytogenes* may undergo bacteriolysis *in vivo* more than in BMMs [32,33]. In mice, we surmise that increased bacterial killing leads to release of nucleic acids and lipoproteins that are sensed by endosomal TLRs and TLR2, respectively. Therefore, secretion of IL-10 in mice is likely dependent on lipoprotein-dependent TLR2 activation and Unc93b1-dependent nucleic-acid sensing TLRs because of increased bacteriolysis in mice.

It was shown previously that administration of anti-IL-10 receptor blocking antibody restored the protective potential of Δ*hly*. However, in this study we observed that, in mice with a defect in endosomal TLR signaling, vaccination with Δ*hly L*. *monocytogenes* did not induce IL-10 secretion but still did not lead to the generation of a protective immune response. A proinflammatory response was also lacking in Unc93b1 mutant mice following vaccination with Δ*hly*, which may explain why a protective immune response was not generated. The differences in immunity following vaccination of WT mice with Δ*hly* and anti-IL10R blocking antibody and vaccination of Unc93b1 mutant mice with Δ*hly* may reflect the fact that anti-IL10R blocking antibody only blocks IL-10 signaling, while mice with defects in endosomal TLR signaling may not be able to generate a proinflammatory response to Δ*hly* and are thus inhibited in generating protective immunity.

Although the results of our study suggest that recognition of bacterial lipoproteins by TLR2 is not the only source of IL-10 *in vivo*, TLR2 remains an attractive pattern recognition receptor to consider for the development of bacterial vaccine delivery systems. *L*. *monocytogenes* expresses over 30 diacylated lipoproteins that are recognized by TLR2/TLR6 dimers [34–37]. Deletion of *lgt* in WT *L*. *monocytogenes* results in delayed secretion of proinflammatory cytokines, but only a modest virulence defect in mice [38]. Thus, removing lipoproteins is a promising strategy for improving *L*. *monocytogenes-*based vaccines by changing the cytokine response without significantly affecting bacterial fitness. One potential method of modulating the response to lipoproteins that has not been explored in *L*. *monocytogenes* is engineering *L*. *monocytogenes* lipoproteins with different acylation states. Triacylated lipoproteins are recognized by TLR2/TLR1 heterodimers and elicit a different inflammatory response than diacylated lipoproteins [39]. More recently, three additional classes of lipoprotein modification with differing abilities to stimulate TLR2 have been described in Gram-positive bacteria [34]. It would be interesting to investigate the contributions of differently acylated lipoproteins to the development of adaptive immunity. Modulating the expression of individual lipoproteins could also impact the immune response. It is not clear whether all *L*. *monocytogenes* lipoproteins contribute to TLR2 activation, or whether *L*. *monocytogenes* has a subset of lipoproteins that specifically activate TLR2. Though the lipid portion of lipoproteins mediates binding of lipoproteins to TLR2, the sequence of the attached peptides affects the ability of lipoproteins to differentially stimulate cytokine secretion [40]. Therefore, changing the expression of specific lipoproteins could be a strategy for fine-tuning the proinflammatory immune response. In addition, TLR2 is traditionally considered a cell-surface-localized TLR, but there is evidence that lipoproteins can activate TLR2 signaling in the endosomal compartment and that signaling from the endosomal compartment can have different effects than from the plasma membrane [41–45]. How specific lipoproteins and acylation state contribute to activation of TLR2, and how TLR2 activation on the cell surface versus in the lysosome contributes to the cytokine response is relevant for the development of bacterial vaccine vectors and warrants further study.

The results of this study indicated that, in mice, TLR2 and endosomal TLRs sense vacuole-confined *L*. *monocytogenes*, resulting in the secretion of IL-10. In our genetic screen, we identified mutants that had increased IL-10 secretion in WT, TLR2^-/-^, and Unc93b1^3d/3d^ macrophages (S2 Table), including transposon insertions in *pgdA* and *oatA*, genes for which mutations have previously been shown to confer increased lysozyme sensitivity [46]. Enhanced IL-10 expression in response to *L*. *monocytogenes* mutants was dependent on both TLR2 and endosomal TLRs, as there were no mutants that induced noteworthy IL-10 secretion in TLR2^-/-^TLR5^-/-^Unc93b1^3d/3d^ BMMs (S2 Table), suggesting that bacterial lysis releases nucleic acids and lipoproteins that are both sensed by the cell. Though we did not observe a decrease in colony forming units of Δ*hly* or the *pgdA* or *oatA* transposon mutants in macrophages (Fig 1D), Δ*hly*Δ*pgdA*Δ*oatA* died rapidly (Fig 2D). Therefore, the individual mutants for which we observed increased IL-10 likely have minor increases in lysis that were not easily detected by CFU. However, in combination, the *pgdA* and *oatA* mutations synergized to yield a mutant that was extremely susceptible to lysis in cells and potently induced IL-10. Importantly, these data suggest that low-level lysis can be detected by TLRs and lead to significant changes in cytokine secretion.

The majority of mutants identified in our screen induced increased IL-10 secretion. Strikingly, while 50 mutants that induced increased IL-10 secretion were identified in our initial screen in Δ*hly*Δ*fla L*. *monocytogenes* (S1 Table), only 4 of those mutants induced significantly increased IL-10 secretion in a Δ*hly* background (S2 Table). It is possible that Δ*hly* may be slightly more sensitive to lysis compared to Δ*hly*Δ*fla*. Flagellar secretion systems require the activity of hydrolases or lytic transglycosylases to degrade peptidoglycan to allow insertion of flagellar components [47,48]. Thus it is reasonable to suspect that the cell wall of Δ*hly* is more fragile than that of Δ*hly*Δ*fla* and is more susceptible to lysis inside vacuoles due to the activity of a flagella-associated peptidoglycan-degrading enzyme. An increase is the basal levels of lysis would increase the basal levels of IL-10 and potentially mask the effects of other mutations that slightly increased lysis. The results of this study suggest that any mutation that promotes bacterial lysis within a phagosome will lead to IL-10 induction.

In this study, we did not investigate whether a particular endosomal nucleic-acid sensing TLR is responsible for induction of IL-10. It is likely that all nucleic-acid sensing TLRs can be activated following bacterial lysis. Bacterial lysis should lead to the release of bacterial contents including: mRNA that can activate TLR7/8, unmethylated CpG chromosomal DNA that can activate TLR9, and ribosomal RNA that can activate TLR13 [49]. Although TLR3 could also recognize double-stranded RNA released upon bacterial lysis, it likely does not to contribute to IL-10 secretion because it was previously demonstrated that IL-10 secretion in response to vacuole-confined *L*. *monocytogenes* is dependent on the signaling adapter MyD88, and TLR3 uses the signaling adapter TRIF [9,49]. It has been previously suggested that bacterial mRNA represents a signature of bacterial viability [50]. Perhaps, then, simultaneous recognition of multiple types of nucleic acid by multiple TLRs is indicative of nonviable bacteria.

The observation that the immune system responds more robustly to bacteria that are alive compared to bacteria that are dead led to the idea that the immune system has ways of monitoring bacterial viability [50–52]. Many signals have been proposed to be “PAMPs per vita” or “vita-PAMPs”—signatures of microbial viability. DNA and RNA have been proposed as possible “PAMP postmortem” (PAMP-PM). In line with the idea of recognition of postmortem PAMPs, the secretion of IL-10 following infection of mice with Δ*hly* could represent a strategy to prevent an unnecessary immune response to bacteria that are already dead, and thus do not pose a threat. To that end, nucleic acid-sensing TLRs are better suited for assessing bacterial viability than TLR2, because living bacteria do not normally release nucleic acids, especially chromosomal DNA, into the surrounding environment. Signaling through nucleic-acid sensing TLRs thus more accurately indicates that a bacterium is dead than sensing of lipoproteins, which can be detected whether a pathogen is alive or dead.

Induction of IL-10 secretion is an important factor to consider in the development of bacterial vaccine vectors. Both live and dead bacterial vaccine vectors have the potential to induce IL-10 secretion. The kinetics of IL-10 secretion may play an important role in determining whether a vaccine will be effective or not. In our study, we identified sensing of nucleic acids as the primary signal for IL-10 induction. For the development of future vaccine strains, strategies to minimize IL-10 induction and immune suppression should be considered. For example, constructs that modify the cell wall could be employed to reduce bacteriolysis in phagosomes. Also, as suggested above, simply deleting flagellin may decrease bacteriolysis. Mutations could also be made to alter lipoproteins and enhance TLR2 activation and proinflammatory cytokine expression. In combination, modifications that reduce IL-10 secretion and modulate proinflammatory cytokines downstream of TLR2 may yield a vaccine strain that has increased potency.

## Materials and methods

### Strain construction

In-frame deletion of genes was performed using allelic exchange as previously described [53]. Δ*hly*Δ*fla* was generated by deleting *hly* in a Δ*flaA* strain (DP-L5986). Δ*hly*Δ*lgt* was generated by deleting *lgt* in a Δ*hly* strain [54]. Δ*hly*Δ*fla*Δ*lgt* was generated by deleting *lgt* in a Δ*hly*Δ*fla* strain. Δ*hly*Δ*pgdA*Δ*oatA* was generated by deleting *hly* in a Δ*pgdA*Δ*oatA* strain (DP-L5220). Δ*hly*Δ*fla*Δ*lgt* was generated by deleting *lgt* in a Δ*hly*Δ*fla* strain. The *lgt* complemented strains were generated by integrating a pPL2 vector encoding *lgt* under control of the pHyper promoter (pPL2t-*pHyper-lgt*) into the *L*. *monocytogenes* genome and selecting for tetracycline-resistant transconjugates [55]. Strains used in this study are listed in S3 Table.

### Transposon library generation

A transposon library was generated in Δ*hly*Δ*fla* as previously described [56]. Transposon mutations were transduced into Δ*hly* using U153 phage as previously described [57].

### Bone marrow-derived macrophage culture

BMM growth media was prepared using high glucose DMEM (Thermo Fisher Scientific) with 20% Fetal Bovine Serum (Seradigm), 1% L-glutamine (Corning), 1% Sodium pyruvate (Corning), 14mM 2-Mercaptoethanol (Gibco), and 10% 3T3 cell supernatant (from M-CSF-producing 3T3 cells). Macrophages were prepared from the femurs of C57BL/6J mice. Femurs were isolated, sterilized with 70% ethanol, and crushed with a mortar and pestle in BMM growth media. Cells were strained through a 70μM filter and distributed into ten 150-mm non-TC dishes in 30mL BMM culture medium. An additional 30mL BMM culture medium was added at day 3. After cells were incubated for a total of seven days at 37°C with 5% C0_2_, cells were harvested and frozen at -80°C in BMM culture medium with 10% Fetal Bovine Serum and 10% DMSO (Sigma) added.

### Intracellular growth of *L*. *monocytogenes* in BMMs

3 × 10^6^ BMMs were plated in 60 mm non-TC-treated Petri dishes with 14 12mm glass coverslips in each dish. Dishes were infected with 5 x 10^5^ CFU (MOI = 0.17) and intracellular growth curves were performed as described previously [58].

### TLR agonists

For experiments using TLR agonists, cells were incubated with the agonists for the entire duration of the experiment. The sequence of CpG ODN 1668 (Integrated DNA Technologies) is: T*C*C*A*T*G*A*C*G*T*T*C*C*T*G*A*T*G*C*T, with asterisks indicating phosphorothioate modifications. CpG ODN 1668 was used at a final concentration of 10μM. Pam2CSK4 (InvivoGen, Cat. tlrl-pm2s-1) was used at a final concentration of 100ng/mL. LPS (InvivoGen, Cat. tlrl-eklps) was used at a final concentration of 50ng/mL.

### Quantification of IL-10 from BMMs– 96-well format

2.6 x 10^5^ BMMs in 200 μL BMM growth media were seeded into wells of a 96-well plate. Bacteria were grown in 1 mL Brain-Heart Infusion Broth containing 200μg/mL streptomycin in 96-well deep well plates at 3°C. Prior to infection, bacteria were pelleted by centrifugation and resuspended in 1 mL PBS. Wells were infected with 8 μL of resuspended bacteria. Plates were infected in duplicate. 30 minutes post-infection, cells were washed with warm PBS, and BMM growth media with 50 μg/mL gentamicin was added. Supernatants were collected and frozen at -80°C until used for analysis. For quantification of IL-10, Mouse IL-10 DuoSet ELISA (R&D Systems) was performed according to manufacturer’s instructions.

### Quantification of IL-10 from BMMs– 24-well format

6 x 10^5^–7 x 10^5^ BMMs in 500 μL BMM growth media were seeded into wells of a 24-well plate. Bacteria were grown overnight at 30°C at a slant without shaking in 3 mL Brain-Heart Infusion Broth containing 200μg/mL streptomycin. Cultures were then pelleted by centrifugation, and resuspended in phosphate-buffered solution (PBS) to an optical density of 2.0. Wells were infected with 20μL of bacteria, approximately 8 x 10^7^ CFU (MOI = 120). Three wells were infected for each bacterial strain. 30 minutes post-infection, cells were washed with warm PBS, and BMM growth media with 50 μg/mL gentamicin was added. Supernatants were collected and frozen at -80°C until used for analysis. For quantification of IL-10, Mouse IL-10 DuoSet ELISA (R&D Systems) was performed according to manufacturer’s instructions. Data was analyzed using GraphPad Prism.

### Animal use ethics statement

All animal work was done in strict accordance with university regulations. Protocols were reviewed and approved by the Animal Care and Use Committee at the University of California, Berkeley **AUP-2016-05-8811.**

### Mice

C57BL/6J mice were purchased from Jackson Laboratories. TLR2^-/-^, Unc93b1^3d/3d^, and TLR2^-/-^TLR5^-/-^Unc93b1^3d/3d^ were provided by Greg Barton (UC Berkeley) and were bred in our facility. To investigate the combined effects of TLR2 and endosomal TLR mutations, TLR2^-/-^TLR5^-/-^Unc93b1^3d/3d^ were used because TLR2^-/-^Unc93b1^3d/3d^ were not available.

### Quantification of cytokines from serum

Eight-to-twelve week old female mice were injected via the tailvein with 10^8^ CFU of Δ*hly* background strains or 10^5^ CFU Δ*actA*. Bacteria were grown overnight at 30°C at a slant without shaking in Brain-Heart Infusion Broth containing 200μg/mL streptomycin. Bacteria were then backdiluted 1:20 and grown at 37°C shaking for about two hours until they reached an optical density of 0.5. Cultures were pelleted and resuspended in PBS to the appropriate concentration, such that mice were infected with 200μL. Four and twenty-four hours post infection, blood was collected from the submandibular vein into Microtainer tubes with serum separator additive (BD). Blood was left to rest for 30 minutes before tubes were centrifuged and serum was collected. Collected serum was stored at -20°C until analysis. Analysis of cytokines from serum was performed using Mouse Inflammation Cytokine Bead Arrays (BD, Cat. 552364). Data was analyzed using FlowJo.

### Vaccination and immune suppression

Eight-to-twelve week old C57BL/6 female mice were vaccinated intravenously via the tail vein with 10^8^ CFU of Δ*hly* background strains or 10^5^ CFU Δ*actA*. Bacteria were grown overnight at 30°C at a slant without shaking in Brain-Heart Infusion Broth containing 200μg/mL streptomycin. Bacteria were then backdiluted 1:20 and grown at 37°C shaking for about two hours until they reached an optical density of 0.5. Cultures were pelleted and resuspended in PBS to the appropriate concentration, such that mice were vaccinated with 200 μL. Eight weeks post-vaccination, mice were challenged with 5 x 10^4^ CFU WT *L*. *monocytogenes*. Three days post-challenge, mice were euthanized with CO_2_ and cervical dislocation and CFU in the spleens and livers were enumerated.

### Statistical analysis

Data were analyzed using GraphPad Prism 8. * indicates P <0.05; ** indicates P <0.01, *** indicates P <0.001, **** indicates P <0.0001; ns indicates no statistical significance.

## Supporting information

S1 Table Secretion of IL-10 in response to infection with transposon mutants in Δ*hly*Δ*fla* background compared to Δ*hly*Δ*fla*. BMMs were infected with *L*. *monocytogenes* at an MOI of 120 in a 24-well plate format. Infections were performed in triplicate for each strain. IL-10 secretion from BMMs was measured from the supernatants by ELISA. The mean amount of IL-10 secreted in response to infection with each Δ*hly*Δ*fla*-background transposon mutant is reported as a percentage of the mean IL-10 induced by infection with Δ*hly*Δ*fla*.
(DOCX)

S2 Table Secretion of IL-10 in response to infection with transposon mutants in Δ*hly* background compared to Δ*hly*. WT, TLR2^-/-^, TLR2^-/-^TLR5^-/-^Unc93b1^3d/3d,^ and Unc93b1^3d/3d^ BMMs were infected with *L*. *monocytogenes* at an MOI of 120 in a 24-well plate format. Infections were performed in triplicate for each strain. IL-10 secretion from BMMs was measured from the supernatants by ELISA. The mean amount of IL-10 secreted in response to infection with each Δ*hly*-background transposon mutant is reported as a percentage of the mean IL-10 induced by infection with Δ*hly*. ^α^ BMM background. ^μ^ IL-10 secretion <80pg/mL. ^β^Δ*hly* transposon mutant IL-10 values were compared to Δ*hly* IL-10 values using Dunnett’s multiple comparisons test, and asterisks indicate level of significance.
(DOCX)

S3 Table *L. monocytogenes* and *E. coli* strains used in this study. (DOCX)
