# Supplementary material for: TLR2 and endosomal TLR-mediated secretion of IL-10 and immune suppression in response to phagosome-confined Listeria monocytogenes
Source: PLoS Pathog. 2020 Jul 7;16(7):e1008622. doi: 10.1371/journal.ppat.1008622 (PMC7340287; doi:10.1371/journal.ppat.1008622)
Supplement: S1 Table — BMMs were infected with L. monocytogenes at an MOI of 120 in a 24-well plate format. Infections were performed in triplicate for each strain. IL-10 secretion from BMMs was measured from the supernatants by ELISA. The mean amount of IL-10 secreted in response to infection with each ΔhlyΔfla-background transposon mutant is reported as a percentage of the mean IL-10 induced by infection with ΔhlyΔfla. (DOCX) [file ppat.1008622.s001.docx]

S1 Table. IL-10 secretion from BMMs infected with transposon mutants in Δ*hly*Δ*fla* background compared to Δ*hly*Δ*fla*.

| Lmo | Gene Annotation | Mean IL-10 (% Δ*hly*Δ*fla* IL-10*)* | Standard Deviation |
| --- | --- | --- | --- |
| *95* | promoter region of lmo0095 | 162 | 14 |
| *286* | pyridoxal phosphate-dependent aminotransferase | 202 | 25 |
| *331* | LPXTG-motif cell wall anchor domain-containing protein | 137 | 6 |
| *333* | inlI, LPXTG-motif cell wall anchor domain-containing protein | 165 | 28 |
| *367* | deferrochelatase/peroxidase EfeB | 142 | 7 |
| *371* | GntR family transcriptional regulator | 77 | 8 |
| 415 | Peptidoglycan/xylan/chitin deacetylase, PgdA/CDA1 family | 172 | 8 |
| 497 | Glycosyl transferase family 2 | 151 | 24 |
| *524* | sulfate transporter | 134 | 1 |
| 580 | Phospholipase/Carboxylesterase | 184 | 7 |
| *635* | HAD family hydrolase | 153 | 17 |
| lmo671-lmo672 | Uncharacterized membrane protein YhaH, DUF805 family | 153 | 12 |
| 709 | Hypothetical protein | 68 | 4 |
| *769* | alpha-1,6-mannanase | 226 | 37 |
| *785* | sigma54-associated activator ManR | 131 | 2 |
| *842* | putative peptidoglycan bound protein (LPXTG motif) | 171 | 28 |
| *848* | amino acid ABC transporter ATP-binding protein | 171 | 19 |
| *954* | promoter region of lmo0954 | 73 | 6 |
| *1080* | teichoic acid biosynthesis protein GgaB | 64 | 13 |
| *1131* | ABC transporter ATP-binding protein | 245 | 14 |
| *1140* | hypothetical protein | 161 | 1 |
| *1241* | hypothetical protein | 158 | 10 |
| *1291* | (oatA) acetyltransferase to YrhL | 158 | 15 |
| *1293* | glycerol-3-phosphate dehydrogenase, glpD | 77 | 8 |
| *1296* | GTPase HflX | 142 | 16 |
| *1366* | 23S rRNA (cytidine1920-2'-O)/16S rRNA (cytidine1409-2'-O)-methyltransferase | 135 | 2 |
| *1395* | protein RodZ, contains Xre-like HTH and DUF4115 domains | 126 | 4 |
| *1429* | energy-coupled thiamine transporter ThiT | 125 | 9 |
| *1499* | endolytic transglycosylase MltG | 153 | 26 |
| *1652* | multidrug ABC transporter permease/ATP-binding protein | *130* | 7 |
| *1695* | mprF | 145 | 17 |
| *1742* | adeC | 154 | 25 |
| *1775* | promoter region, purE, phosphoribosylaminoimidazole carboxylase catalytic subunit | 144 | 2 |
| *1799* | putative peptidoglycan bound protein (LPXTG motif) | 252 | 11 |
| *1835* | pyrAB | 213 | 29 |
| *1843* | RluA family pseudouridine synthase | 153 | 19 |
| *1877* | formyl-tetrahydrofolate synthetase | 151 | 11 |
| *1956* | Fur family transcriptional regulator, ferric uptake regulator | 77 | 3 |
| *2027* | putative cell surface protein, similar to internalin proteins | 198 | 10 |
| 2079 | hypothetical protein | 78 | 8 |
| *2128* | transcriptional regulator, LacI family | 178 | 11 |
| 2229 | penicillin-binding protein 2A | 141 | 10 |
| *2287* | putative tape-measure [Bacteriophage A118] | 183 | 5 |
| 2389 | NADH dehydrogenase | 146 | 18 |
| *2482* | *(lgt)* prolipoprotein diacylglyceryl transferase | 9 | 1 |
| 2529 | ATP synthase F0F1 subunit beta | 159 | 22 |
| *2530* | atpG | 146 | 27 |
| *2531* | ATP synthase F0F1 subunit alpha | 16 | 2 |
| *2581* | promoter region of ABC transporter permease | 154 | 13 |
| *2634* | energy-coupling factor transporter transmembrane protein EcfT | 164 | 23 |
| 2634 | energy-coupling factor transporter transmembrane protein EcfT | 135 | 8 |
| *2641* | heptaprenyl diphosphate synthase | 163 | 19 |
| *2720* | acyl--CoA ligase | 161 | 29 |
| *2757* | DNA helicase RecQ | 152 | 5 |
| *2760* | ABC transporter ATP-binding protein | 174 | 19 |
| 2816 | Sugar phosphate permease | 132 | 11 |
| *2835* | xylose isomerase | 172 | 20 |
| *2835* | xylose isomerase | 165 | 1 |
| *2854* | Membrane protein insertase YidC 2 | 156 | 30 |
