# Supplementary material for: TLR2 and endosomal TLR-mediated secretion of IL-10 and immune suppression in response to phagosome-confined Listeria monocytogenes
Source: PLoS Pathog. 2020 Jul 7;16(7):e1008622. doi: 10.1371/journal.ppat.1008622 (PMC7340287; doi:10.1371/journal.ppat.1008622)
Supplement: S2 Table — WT, TLR2-/-, TLR2-/-TLR5-/-Unc93b13d/3d, and Unc93b13d/3d BMMs were infected with L. monocytogenes at an MOI of 120 in a 24-well plate format. Infections were performed in triplicate for each strain. IL-10 secretion from BMMs was measured from the supernatants by ELISA. The mean amount of IL-10 secreted in response to infection with each Δhly-background transposon mutant is reported as a percentage of the mean IL-10 induced by infection with Δhly. α BMM background. μ IL-10 secretion <80pg/mL. βΔhly transposon mutant IL-10 values were compared to Δhly IL-10 values using Dunnett’s multiple comparisons test, and asterisks indicate level of significance. (DOCX) [file ppat.1008622.s002.docx]

S2 Table. IL-10 secretion from BMMs infected with transposon mutants in Δ*hly* background compared to Δ*hly*.

| Lmo | Gene Annotation | WT^α^ | Sign.^β^ | TLR2^α^ | Sign.^β^ | Unc93b1^3d/3d α^ | Sign.^β^ | TLR2^-/-^TLR5^-/-^Unc93b1^3d/3d α^ |
| --- | --- | --- | --- | --- | --- | --- | --- | --- |
| *5* | promoter region of lmo0095 | 88 | ns | 114 | ns | 125 | ns | µ |
| *286* | pyridoxal phosphate-dependent aminotransferase | 118 | ns | 96 | ns | 105 | ns | µ |
| *331* | LPXTG-motif cell wall anchor domain-containing protein | 97 | ns | 131 | ns | 128 | ns | µ |
| *333* | inlI, LPXTG-motif cell wall anchor domain-containing protein | 119 | ns | 111 | ns | 130 | ns | µ |
| *367* | deferrochelatase/peroxidase EfeB | 116 | ns | 134 | ns | 142 | ns | µ |
| *371* | GntR family transcriptional regulator | 101 | ns | 177 | ** | 130 | * | µ |
| 415 | Peptidoglycan/xylan/chitin deacetylase, PgdA/CDA1 family | 177 | **** | 493 | **** | 174 | **** | µ |
| 497 | Glycosyl transferase family 2 | 89 | ns | 108 | ns | 116 | ns | µ |
| *524* | sulfate transporter | 108 | ns | 136 | ns | 155 | * | µ |
| 580 | Phospholipase/Carboxylesterase | 96 | ns | 131 | ns | 115 | ns | µ |
| *635* | HAD family hydrolase | 127 | ns | 139 | ns | 142 | ns | µ |
| lmo671-lmo672 | Uncharacterized membrane protein YhaH, DUF805 family | 104 | ns | 128 | ns | 117 | ns | µ |
| *709* | hypothetical protein | 41 | **** | 64 | ns | 77 | ns | µ |
| *769* | alpha-1,6-mannanase | 133 | ns | 185 | ns | 144 | ** | µ |
| *785* | sigma54-associated activator ManR | 120 | ns | 118 | ns | 128 | ns | µ |
| *842* | putative peptidoglycan bound protein (LPXTG motif) | 170 | ns | 153 | ns | 175 | **** | µ |
| *848* | amino acid ABC transporter ATP-binding protein | 85 | ns | 90 | ns | 112 | ns | µ |
| *954* | promoter region of lmo0954 | 90 | ns | 100 | ns | 113 | ns | µ |
| *1131* | ABC transporter ATP-binding protein | 114 | ns | 148 | ns | 152 | *** | µ |
| *1140* | hypothetical protein | 124 | ns | 106 | ns | 124 | ns | µ |
| *1241* | hypothetical protein | 109 | ns | 135 | ns | 121 | ns | µ |
| *1291* | (oatA) acetyltransferase to YrhL | 139 | ** | 327 | **** | 184 | **** | µ |
| *1293* | glycerol-3-phosphate dehydrogenase, glpD | 122 | ns | 137 | ns | 109 | ns | µ |
| *1296* | GTPase HflX | 138 | ns | 121 | ns | 143 | * | µ |
| *1366* | 23S rRNA (cytidine1920-2'-O)/16S rRNA (cytidine1409-2'-O)-methyltransferase | 102 | ns | 128 | ns | 145 | * | µ |
| *1395* | protein RodZ, contains Xre-like HTH and DUF4115 domains | 91 | ns | 113 | ns | 146 | ns | µ |
| *1429* | energy-coupled thiamine transporter ThiT | 124 | ns | 184 | ** | 127 | ns | µ |
| *1499* | endolytic transglycosylase MltG | 114 | ns | 137 | ns | 115 | ns | µ |
| *1652* | multidrug ABC transporter permease/ATP-binding protein | 97 | ns | 148 | * | 148 | ns | µ |
| *1695* | mprF | 99 | ns | 86 | ns | 179 | *** | µ |
| *1742* | adeC | 126 | ns | 152 | ns | 176 | *** | µ |
| *1775* | promoter region, purE, phosphoribosylaminoimidazole carboxylase catalytic subunit | 107 | ns | 200 | *** | 136 | ns | µ |
| *1799* | putative peptidoglycan bound protein (LPXTG motif) | 108 | ns | 134 | ns | 137 | ns | µ |
| *1835* | pyrAB | 135 | ns | 103 | ns | 165 | **** | µ |
| *1843* | RluA family pseudouridine synthase | 127 | ns | 154 | ns | 124 | ns | µ |
| *1877* | formyl-tetrahydrofolate synthetase | 125 | ns | 133 | ns | 123 | ns | µ |
| *1956* | Fur family transcriptional regulator, ferric uptake regulator | 97 | ns | 207 | **** | 129 | ns | µ |
| *2027* | putative cell surface protein, similar to internalin proteins | 118 | ns | 147 | * | 114 | ns | µ |
| 2079 | hypothetical protein | 73 | ns | 139 | ns | 131 | ns | µ |
| *2128* | transcriptional regulator, LacI family | 119 | ns | 133 | ns | 131 | * | µ |
| 2229 | penicillin-binding protein 2A | 122 | ns | 133 | ns | 148 | **** | µ |
| *2287* | putative tape-measure [Bacteriophage A118] | 114 | ns | 108 | ns | 132 | ns | µ |
| 2389 | NADH dehydrogenase | 88 | ns | 162 | ns | 112 | ns | µ |
| *2482* | *(lgt)* prolipoprotein diacylglyceryl transferase | 27 | **** | 162 | ** | 4 | **** | µ |
| 2529 | ATP synthase F0F1 subunit beta | 158 | * | 221 | **** | 162 | ** | µ |
| *2530* | atpG | 209 | ns | 251 | **** | 118 | ns | µ |
| *2531* | ATP synthase F0F1 subunit alpha | 113 | ns | 212 | *** | 106 | ns | µ |
| *2581* | promoter region of ABC transporter permease | 96 | ns | 177 | * | 160 | *** | µ |
| 2634 | energy-coupling factor transporter transmembrane protein EcfT | 140 | * | 145 | * | 154 | ** | µ |
| *2634* | energy-coupling factor transporter transmembrane protein EcfT | 185 | ns | 342 | * | 158 | ** | µ |
| *2641* | heptaprenyl diphosphate synthase | 95 | ns | 194 | *** | 135 | ns | µ |
| *2720* | acyl--CoA ligase | 89 | ns | 105 | ns | 113 | ns | µ |
| *2757* | DNA helicase RecQ | 108 | ns | 105 | ns | 156 | ns | µ |
| *2760* | ABC transporter ATP-binding protein | 95 | ns | 100 | ns | 124 | ns | µ |
| 2816 | Sugar phosphate permease | 102 | ns | 170 | ns | 116 | ns | µ |
| *2835* | xylose isomerase | 90 | ns | 151 | ns | 109 | ns | µ |
| *2854* | Membrane protein insertase YidC 2 | 136 | ns | 134 | ns | 167 | ns | µ |
