# Supplementary material for: TLR2 and endosomal TLR-mediated secretion of IL-10 and immune suppression in response to phagosome-confined Listeria monocytogenes
Source: PLoS Pathog. 2020 Jul 7;16(7):e1008622. doi: 10.1371/journal.ppat.1008622 (PMC7340287; doi:10.1371/journal.ppat.1008622)
Supplement: S3 Table — (DOCX) [file ppat.1008622.s003.docx]

S3 Table. List of Strains.

| **Strain name** | **Strain background** | **Lmo of Gene with Transposon Insertion** | **Integrated Plasmid** | **Strain Number** | **Reference** |
| --- | --- | --- | --- | --- | --- |
| Δ*hly* | Δ*hly* | - | - | DP-L2161 | [54] |
| Δ*flaA* | Δ*flaA* |  |  | DP-L5986 | This study |
| *hly* knockout plasmid | *E. coli* | - | - | DP-L2154 | [54] |
| Δ*hly*Δ*fla* | Δ*hly*Δ*fla* | - | - | DP-L7001 | This study |
| *lgt* knockout plasmid | *E. coli* | - | - | DP-E7002 | This study |
| Δ*hly*Δ*lgt* | Δ*hly*Δ*lgt* | - | - | DP-L7003 | This study |
| Δ*pgdA*Δ*oatA* | Δ*pgdA*Δ*oatA* | - | - | DP-L5220 | [26] |
| Δ*hly*Δ*pgdA*Δ*oatA* | Δ*hly*Δ*pgdA*Δ*oatA* | - | - | DP-L7004 | This study |
| Δ*hly*Δ*fla*Δ*lgt* | Δ*hly*Δ*fla*Δ*lgt* | - | - | DP-L7005 | This study |
| pPL2t-*pHyper-lgt* | *E. coli* | - | - | DP-E7006 | This study |
| Δ*hly*Δ*lgt* + *lgt* | Δ*hly*Δ*lgt* | - | pPL2t-*pHyper-lgt* | DP-L7007 | This study |
| Δ*hly*Δ*fla*Δ*lgt* + *lgt* | Δ*hly*Δ*fla*Δ*lgt* | - | pPL2t-*pHyper-lgt* | DP-L7008 | This study |
| ΔactA | ΔactA | - | - | DP-L4029 | [55] |
|  | Δ*hly*Δ*fla* | *Lmo0095* |  | DP-L7009 | This study |
|  | Δ*hly*Δ*fla* | *Lmo0286* |  | DP-L7010 | This study |
|  | Δ*hly*Δ*fla* | *Lmo0331* |  | DP-L7011 | This study |
|  | Δ*hly*Δ*fla* | *Lmo0333* |  | DP-L7012 | This study |
|  | Δ*hly*Δ*fla* | *Lmo0367* |  | DP-L7013 | This study |
|  | Δ*hly*Δ*fla* | *Lmo0371* |  | DP-L7014 | This study |
|  | Δ*hly*Δ*fla* | *Lmo0*415 |  | DP-L7015 | This study |
|  | Δ*hly*Δ*fla* | *Lmo0*497 |  | DP-L7016 | This study |
|  | Δ*hly*Δ*fla* | *Lmo0524* |  | DP-L7017 | This study |
|  | Δ*hly*Δ*fla* | *Lmo0*580 |  | DP-L7018 | This study |
|  | Δ*hly*Δ*fla* | *Lmo0635* |  | DP-L7019 | This study |
|  | Δ*hly*Δ*fla* | *Intergenic;*  *Lmo0*671- *Lmo0*672 |  | DP-L7020 | This study |
|  | Δ*hly*Δ*fla* | *Lmo0*709 |  | DP-L7021 | This study |
|  | Δ*hly*Δ*fla* | *Lmo0769* |  | DP-L7022 | This study |
|  | Δ*hly*Δ*fla* | *Lmo0785* |  | DP-L7023 | This study |
|  | Δ*hly*Δ*fla* | *Lmo0842* |  | DP-L7024 | This study |
|  | Δ*hly*Δ*fla* | *Lmo0848* |  | DP-L7025 | This study |
|  | Δ*hly*Δ*fla* | *Lmo0954* |  | DP-L7026 | This study |
|  | Δ*hly*Δ*fla* | *Lmo1080* |  | DP-L7027 | This study |
|  | Δ*hly*Δ*fla* | *Lmo1131* |  | DP-L7028 | This study |
|  | Δ*hly*Δ*fla* | *Lmo1140* |  | DP-L7029 | This study |
|  | Δ*hly*Δ*fla* | *Lmo1241* |  | DP-L7030 | This study |
|  | Δ*hly*Δ*fla* | *Lmo1291* |  | DP-L7031 | This study |
|  | Δ*hly*Δ*fla* | *Lmo1293* |  | DP-L7032 | This study |
|  | Δ*hly*Δ*fla* | *Lmo1296* |  | DP-L7033 | This study |
|  | Δ*hly*Δ*fla* | *Lmo1366* |  | DP-L7034 | This study |
|  | Δ*hly*Δ*fla* | *Lmo1395* |  | DP-L7035 | This study |
|  | Δ*hly*Δ*fla* | *Lmo1429* |  | DP-L7036 | This study |
|  | Δ*hly*Δ*fla* | *Lmo1499* |  | DP-L7037 | This study |
|  | Δ*hly*Δ*fla* | *Lmo1652* |  | DP-L7038 | This study |
|  | Δ*hly*Δ*fla* | *Lmo1695* |  | DP-L7039 | This study |
|  | Δ*hly*Δ*fla* | *Lmo1742* |  | DP-L7040 | This study |
|  | Δ*hly*Δ*fla* | *Lmo1775* |  | DP-L7041 | This study |
|  | Δ*hly*Δ*fla* | *Lmo1799* |  | DP-L7042 | This study |
|  | Δ*hly*Δ*fla* | *Lmo1835* |  | DP-L7043 | This study |
|  | Δ*hly*Δ*fla* | *Lmo1843* |  | DP-L7044 | This study |
|  | Δ*hly*Δ*fla* | *Lmo1877* |  | DP-L7045 | This study |
|  | Δ*hly*Δ*fla* | *Lmo1956* |  | DP-L7046 | This study |
|  | Δ*hly*Δ*fla* | *Lmo2027* |  | DP-L7047 | This study |
|  | Δ*hly*Δ*fla* | *Lmo2079* |  | DP-L7048 | This study |
|  | Δ*hly*Δ*fla* | *Lmo2128* |  | DP-L7049 | This study |
|  | Δ*hly*Δ*fla* | *Lmo2229* |  | DP-L7050 | This study |
|  | Δ*hly*Δ*fla* | *Lmo2287* |  | DP-L7051 | This study |
|  | Δ*hly*Δ*fla* | *Lmo2389* |  | DP-L7052 | This study |
|  | Δ*hly*Δ*fla* | *Lmo2482* |  | DP-L7053 | This study |
|  | Δ*hly*Δ*fla* | *Lmo2529* |  | DP-L7054 | This study |
|  | Δ*hly*Δ*fla* | *Lmo2530* |  | DP-L7055 | This study |
|  | Δ*hly*Δ*fla* | *Lmo2531* |  | DP-L7056 | This study |
|  | Δ*hly*Δ*fla* | *Lmo2581* |  | DP-L7057 | This study |
|  | Δ*hly*Δ*fla* | *Lmo2634* |  | DP-L7058 | This study |
|  | Δ*hly*Δ*fla* | *Lmo2634* |  | DP-L7059 | This study |
|  | Δ*hly*Δ*fla* | *Lmo2641* |  | DP-L7060 | This study |
|  | Δ*hly*Δ*fla* | *Lmo2720* |  | DP-L7061 | This study |
|  | Δ*hly*Δ*fla* | *Lmo2757* |  | DP-L7062 | This study |
|  | Δ*hly*Δ*fla* | *Lmo2760* |  | DP-L7063 | This study |
|  | Δ*hly*Δ*fla* | *Lmo2816* |  | DP-L7064 | This study |
|  | Δ*hly*Δ*fla* | *Lmo2835* |  | DP-L7065 | This study |
|  | Δ*hly*Δ*fla* | *Lmo2835* |  | DP-L7066 | This study |
|  | Δ*hly*Δ*fla* | *Lmo2854* |  | DP-L7067 | This study |
|  | Δ*hly* | *Lmo0095* |  | DP-L7068 | This study |
|  | Δ*hly* | *Lmo0286* |  | DP-L7069 | This study |
|  | Δ*hly* | *Lmo0331* |  | DP-L7070 | This study |
|  | Δ*hly* | *Lmo0333* |  | DP-L7071 | This study |
|  | Δ*hly* | *Lmo0367* |  | DP-L7072 | This study |
|  | Δ*hly* | *Lmo0371* |  | DP-L7073 | This study |
| Δ*hly pgdA::*Tn | Δ*hly* | *Lmo0*415 |  | DP-L7074 | This study |
|  | Δ*hly* | *Lmo0*497 |  | DP-L7075 | This study |
|  | Δ*hly* | *Lmo0524* |  | DP-L7076 | This study |
|  | Δ*hly* | *Lmo0*580 |  | DP-L7077 | This study |
|  | Δ*hly* | *Lmo0635* |  | DP-L7078 | This study |
|  | Δ*hly* | *Intergenic;*  *Lmo0*671- *Lmo0*672 |  | DP-L7079 | This study |
| Δ*hly lmo0709::*Tn | Δ*hly* | *Lmo0*709 |  | DP-L7080 | This study |
|  | Δ*hly* | *Lmo0769* |  | DP-L7081 | This study |
|  | Δ*hly* | *Lmo0785* |  | DP-L7082 | This study |
|  | Δ*hly* | *Lmo0842* |  | DP-L7083 | This study |
|  | Δ*hly* | *Lmo0848* |  | DP-L7084 | This study |
|  | Δ*hly* | *Lmo0954* |  | DP-L7085 | This study |
|  | Δ*hly* | *Lmo1131* |  | DP-L7086 | This study |
|  | Δ*hly* | *Lmo1140* |  | DP-L7087 | This study |
|  | Δ*hly* | *Lmo1241* |  | DP-L7088 | This study |
| Δ*hly oatA::*Tn | Δ*hly* | *Lmo1291* |  | DP-L7089 | This study |
|  | Δ*hly* | *Lmo1293* |  | DP-L7090 | This study |
|  | Δ*hly* | *Lmo1296* |  | DP-L7091 | This study |
|  | Δ*hly* | *Lmo1366* |  | DP-L7092 | This study |
|  | Δ*hly* | *Lmo1395* |  | DP-L7093 | This study |
|  | Δ*hly* | *Lmo1429* |  | DP-L7094 | This study |
|  | Δ*hly* | *Lmo1499* |  | DP-L7095 | This study |
|  | Δ*hly* | *Lmo1652* |  | DP-L7096 | This study |
|  | Δ*hly* | *Lmo1695* |  | DP-L7097 | This study |
|  | Δ*hly* | *Lmo1742* |  | DP-L7098 | This study |
|  | Δ*hly* | *Lmo1775* |  | DP-L7099 | This study |
|  | Δ*hly* | *Lmo1799* |  | DP-L7100 | This study |
|  | Δ*hly* | *Lmo1835* |  | DP-L7101 | This study |
|  | Δ*hly* | *Lmo1843* |  | DP-L7102 | This study |
|  | Δ*hly* | *Lmo1877* |  | DP-L7103 | This study |
|  | Δ*hly* | *Lmo1956* |  | DP-L7104 | This study |
|  | Δ*hly* | *Lmo2027* |  | DP-L7105 | This study |
|  | Δ*hly* | *Lmo2079* |  | DP-L7106 | This study |
|  | Δ*hly* | *Lmo2128* |  | DP-L7107 | This study |
|  | Δ*hly* | *Lmo2229* |  | DP-L7108 | This study |
|  | Δ*hly* | *Lmo2287* |  | DP-L7109 | This study |
|  | Δ*hly* | *Lmo2389* |  | DP-L7110 | This study |
| Δ*hly lgt::*Tn | Δ*hly* | *Lmo2482* |  | DP-L7111 | This study |
| Δ*hly lmo2529::*Tn | Δ*hly* | *Lmo2529* |  | DP-L7112 | This study |
|  | Δ*hly* | *Lmo2530* |  | DP-L7113 | This study |
|  | Δ*hly* | *Lmo2531* |  | DP-L7114 | This study |
|  | Δ*hly* | *Lmo2581* |  | DP-L7115 | This study |
| Δ*hly lmo2634::*Tn | Δ*hly* | *Lmo2634* |  | DP-L7116 | This study |
|  | Δ*hly* | *Lmo2634* |  | DP-L7117 | This study |
|  | Δ*hly* | *Lmo2641* |  | DP-L7118 | This study |
|  | Δ*hly* | *Lmo2720* |  | DP-L7119 | This study |
|  | Δ*hly* | *Lmo2757* |  | DP-L7120 | This study |
|  | Δ*hly* | *Lmo2760* |  | DP-L7121 | This study |
|  | Δ*hly* | *Lmo2816* |  | DP-L7122 | This study |
|  | Δ*hly* | *Lmo2835* |  | DP-L7123 | This study |
|  | Δ*hly* | *Lmo2835* |  | DP-L7124 | This study |
|  | Δ*hly* | *Lmo2854* |  | DP-L7125 | This study |
|  |  |  |  |  |  |
